# Supplementary material for: Assessment of Recombinant β-Propeller Phytase of the Bacillus Species Expressed Intracellularly in Yarrowia lipolityca
Source: J Fungi (Basel). 2025 Feb 26;11(3):186. doi: 10.3390/jof11030186 (PMC11943157; doi:10.3390/jof11030186)
Supplement: Supplementary file 1 [file jof-11-00186-s001.zip › jof-3376775-supplementary.pdf]

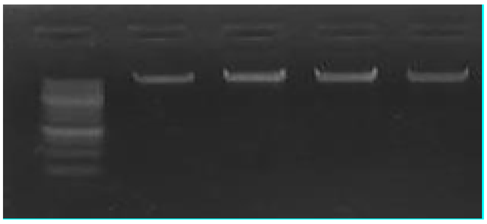

Figure S1. Electrophoresis of total genomic DNA in 1.2% agarose gel. Visualization genomic DNA using a UV transilluminator at a wavelength of 365 nm. Tracks: 1 – DNA marker 1kB; 2 – genomic DNA *B. subtilis* UQM 41285, 3 – *B. cereus* ATCC 11778, 4 – *B.licheniformis var. mycoides* 537, 5 – *B. amyloliquefaciens* (B10986).

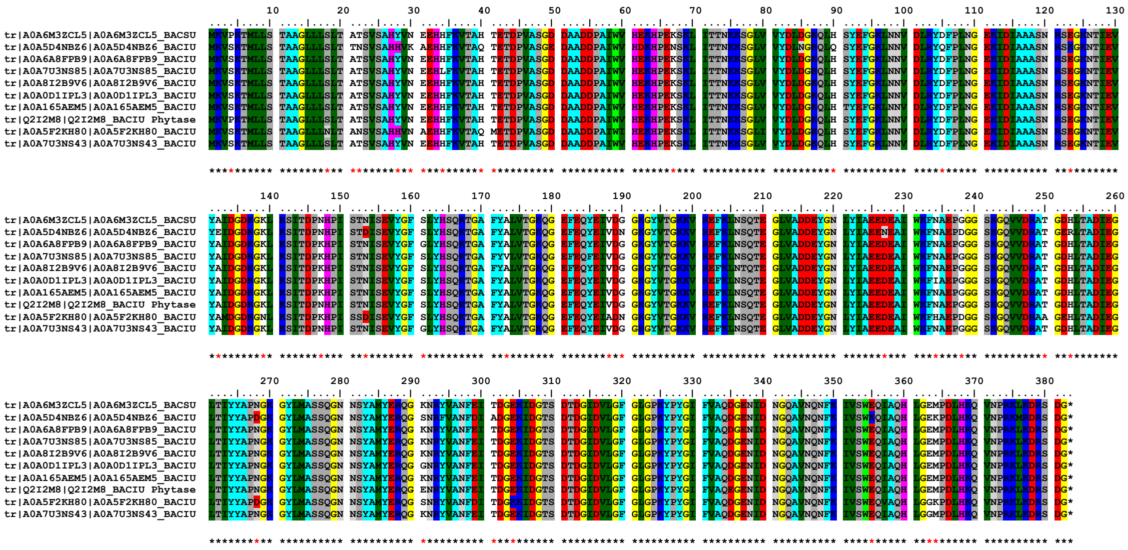

Figure S2 – Comparison (analysis) of amino acid sequences of phytases from the *Bacillus subtilis* strains presented in the NCBI database (https://www.ncbi.nlm.nih.gov/) or uniprot (https://www.uniprot.org/). UniProtKB: A0A6M3ZCL5, genebank:NP_389861.1, UniProtKB: A0A5D4NBZ6, UniProtKB: A0A6A8FPB9, UniProtKB: A0A7U3NS85, UniProtKB: A0A8I2B9V6, UniProtKB: A0A0D1IPL3, UniProtKB: A0A165AEM5, UniProtKB: Q2I2M8, UniProtKB: A0A5F2KH80, UniProtKB: A0A7U3NS43.

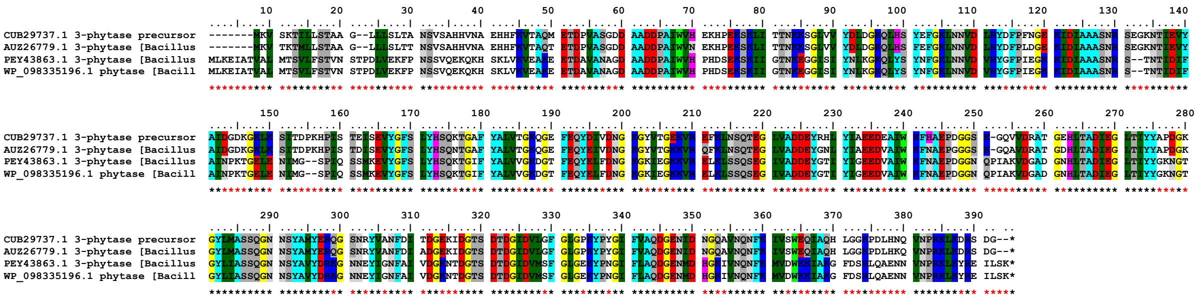

Figure S3. Comparison (analysis) of amino acid sequences of phytases from the *Bacillus cereus* strains in the NCBI database (https://www.ncbi.nlm.nih.gov/) or uniprot (https://www.uniprot.org/). GenBank: CUB29737.1; GenBank: AUZ26779.1; GenBank: PEY43863.1; GenBank: WP_098335196.1

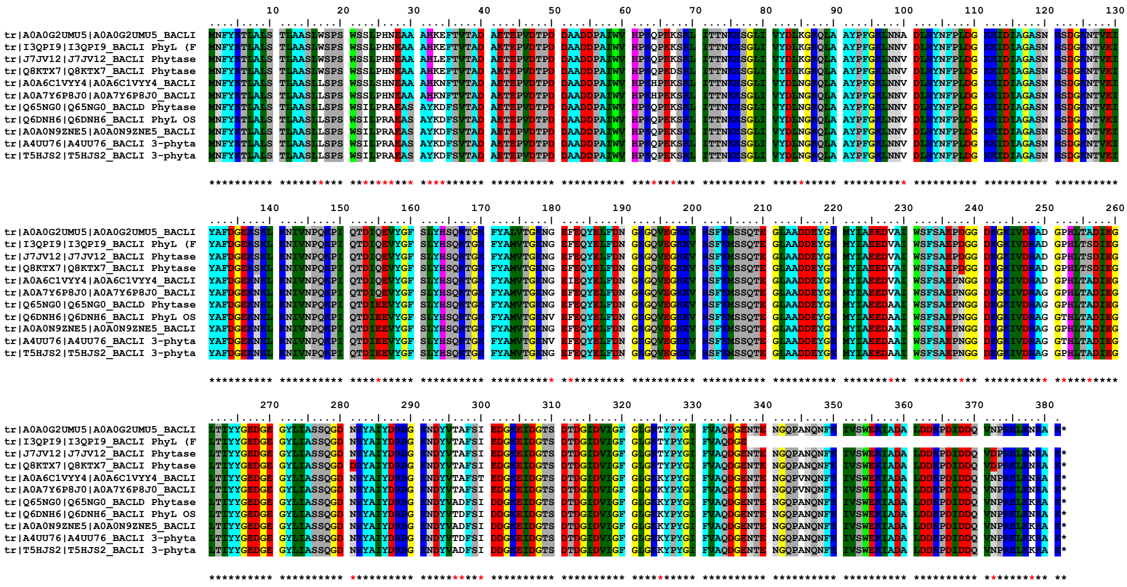

Figure S4 – Comparison (analysis) of amino acid sequences of phytases from the *Bacillus licheniformis* strains presented in the NCBI database (https://www.ncbi.nlm.nih.gov/) or uniprot (https://www.uniprot.org/). UniProtKB: A0A0G2UMU5, UniProtKB: I3QPI9, UniProtKB: J7JV12, UniProtKB: Q8KTX7, UniProtKB: A0A6C1VYY4, UniProtKB: A0A7Y6P8J0, UniProtKB: Q65NG0, UniProtKB: Q6DNH6, UniProtKB: niprotkb: A0A0N9ZNE5, UniProtKB: A4UU76, UniProtKB: T5HJS2.

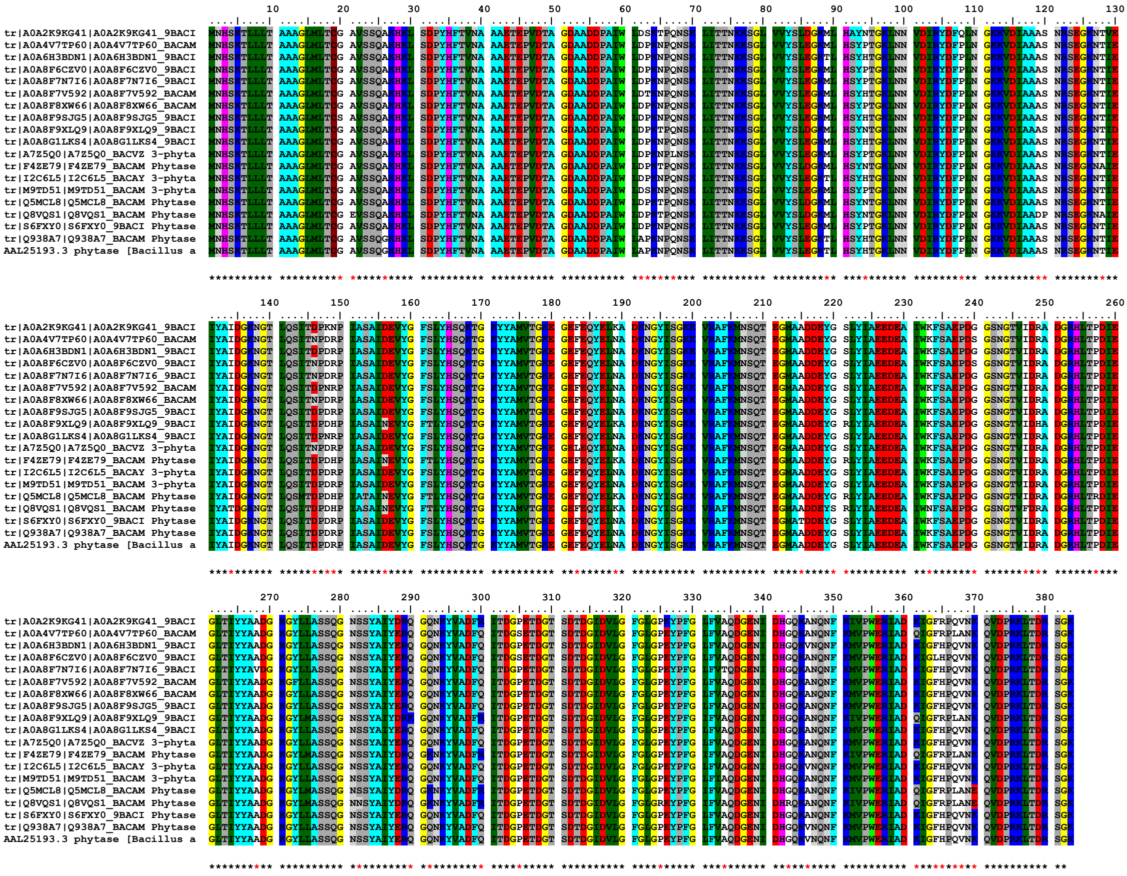

Figure S5 – Comparison (analysis) of amino acid sequences of phytases from the *Bacillus amyloliquefaciens* strains presented in the NCBI database (https://www.ncbi.nlm.nih.gov/) or uniprot (https://www.uniprot.org/). UniProtKB: A0A2K9KG41, UniProtKB: A0A4V7TP60, UniProtKB: A0A6H3BDN1, UniProtKB: A0A8F6CZV0, UniProtKB: A0A8F7N7I6, UniProtKB: A0A8F7V592, UniProtKB: A0A8F9SJG5, UniProtKB: A0A8F9XLQ9, UniProtKB: A0A8G1LKS4, UniProtKB: A7Z5Q0, UniProtKB: F4ZE79, UniProtKB: I2C6L5, UniProtKB: M9TD51, UniProtKB: Q5MCL8, UniProtKB: Q8VQS1, UniProtKB: S6FXY0, UniProtKB: Q938A7, UniProtKB: AAL25193.3.

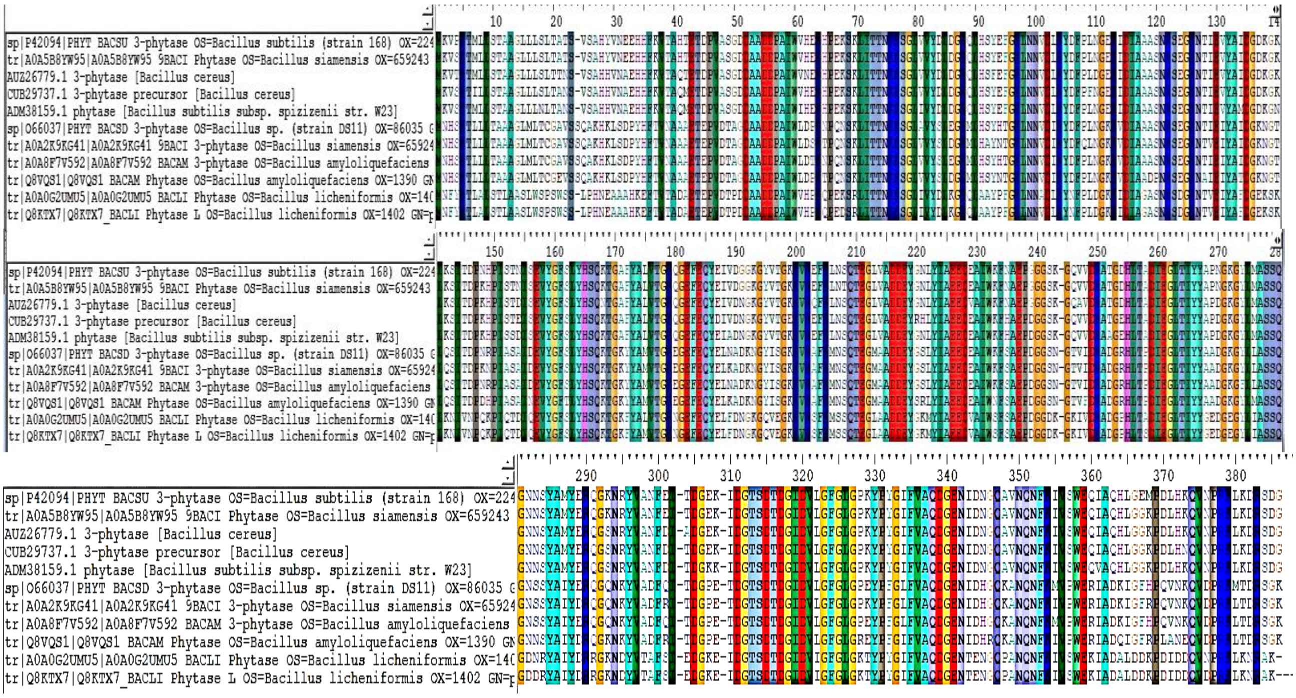

Figure S6 – Comparison (analysis) of amino acid sequences of phytases from the *Bacillus* species in the NCBI database (https://www.ncbi.nlm.nih.gov/) or uniprot (https://www.uniprot.org/). UniProtKB: P42094, genebank: NC_000964; UniProtKB: A0A5B8YW95; UniProtKB: 066037; UniProtKB: AUZ26779.1, UniProtKB: CUB29737.1, UniProtKB: ADM38159.1, UniProtKB: AHM26864.1, UniProtKB: A0A2K9KG41, UniProtKB: A0A8F7V592, UniProtKB: Q8VQS1; UniProtKB: A0A0G2UMU5, UniProtKB: Q8KTX7.

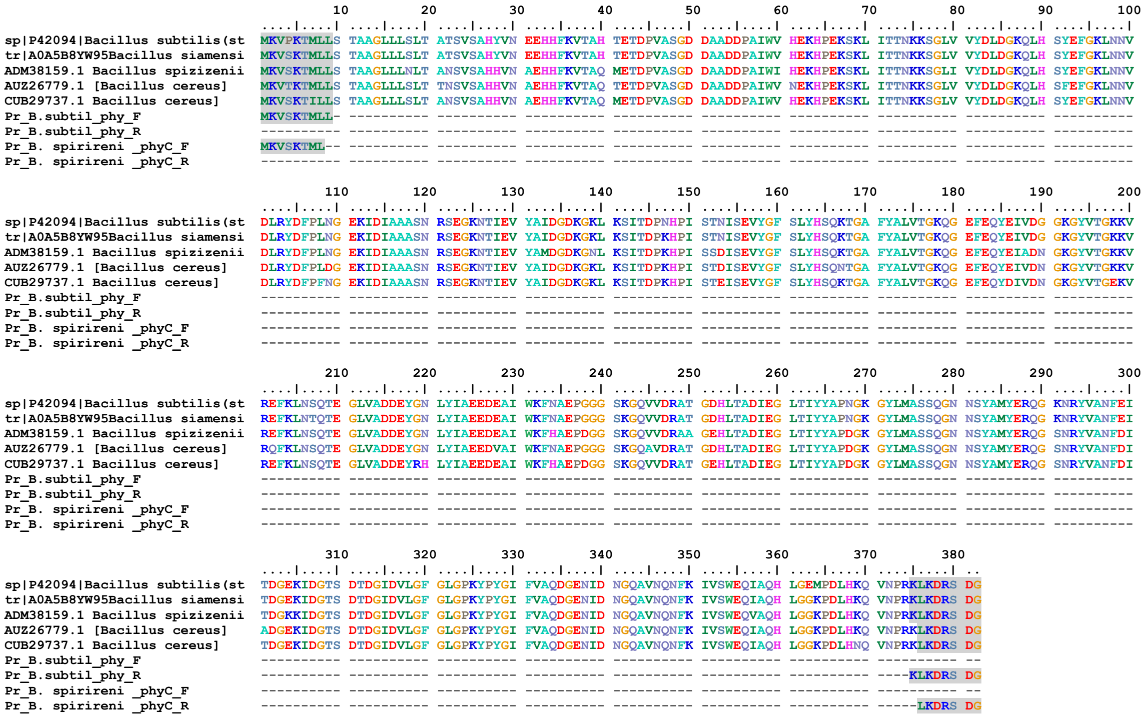

Figure S7. Alignment of amino acid sequences of the PhyD first isoform. Pr_B.subtil_phy_F/Pr_B.subtil_phy_R and Pr_B. spirireni _phyC_F/Pr_B. spirireni _phyC are the locations of the universal primers at the terminal highly conserved regions of the amino acid sequence.

```
                                     10         20         30         40         50         60         70         80         90        100
                               ....|....| ....|....| ....|....| ....|....| ....|....| ....|....| ....|....| ....|....| ....|....| ....|....|
sp|O66037|PHYT_BACSD 3-phytase MNHSKTLLLT AAAGLMLTCG AVSSQAKHKL SDPYHFTVNA AAETEPVDTA GDAADDPAIW LDPKNPQNSK LITTNKKSGL AVYSLEGKML HSYHTGKLNN
tr|A0A2K9KG41|A0A2K9KG41_9BACI MNHSKTLLLT AAAGLMLTCG AVSSQAKHKL SDPYHFTVNA AAETEPVDTA GDAADDPAIW LDSKTPQNSK LITTNKKSGL VVYSLDGKML HAYNTGKLNN
tr|A0A8F7V592|A0A8F7V592_BACAM MNHSKTLLLT AAAGLMLTCG AVSSQAKHKL SDPYHFTVNA AAETEPVDTA GDAADDPAIW LDTKNPQNSK LITTNKKSGL VVYSLEGKML HSYHTGKLNN
tr|Q8VQS1|Q8VQS1_BACAM Phytase MNHSKTLLLT AAAGLMLTCG EVSSQAKHKL SDPYHFTVNA AAETEPVDTA GDAADDPAIW LDPKTPQNSK LITTNKKSGL VVYSLDGKML HSYNTGKLNN
Pr_B.amylo/siam_phyC_F         MNHSKTLLL- ---------- ---------- ---------- ---------- ---------- ---------- ---------- ---------- ----------
Pr_B.amylo/siam_phyC_R         ---------- ---------- ---------- ---------- ---------- ---------- ---------- ---------- ---------- ----------

                                    110        120        130        140        150        160        170        180        190        200
                               ....|....| ....|....| ....|....| ....|....| ....|....| ....|....| ....|....| ....|....| ....|....| ....|....|
sp|O66037|PHYT_BACSD 3-phytase VDIRYDFPLN GKKVDIAAAS NRSEGKNTIE IYAIDGKNGT LQSITDPNRP IASAIDEVYG FSLYHSQKTG KYYAMVTGKE GEFEQYELNA DKNGYISGKK
tr|A0A2K9KG41|A0A2K9KG41_9BACI VDIRYDFQLN GKKVDIAAAS NRSEGKNTVE IYAIDGKNGT LQSITDPKNP IASAIDEVYG FSLYHSQKTG KYYAMVTGKE GEFEQYELKA DKNGYISGKK
tr|A0A8F7V592|A0A8F7V592_BACAM VDIRYDFPLN GKKVDIAAAS NRSEGKNTIE IYAIDGKNGT LQSITDPNRP IASAIDEVYG FSLYHSQKTG KYYAMVTGKE GEFEQYELNA DKNGYISGKK
tr|Q8VQS1|Q8VQS1_BACAM Phytase VDIRYDFPLN GKKVDIAADP NRSEGKNAIE IYATDGKNGT LQSITDPDHP IASAINEVYG FTLYHSQKTG KYYAMVTGKE GEFEQYELKA DKNGYISGKK
Pr_B.amylo/siam_phyC_F         ---------- ---------- ---------- ---------- ---------- ---------- ---------- ---------- ---------- ----------
Pr_B.amylo/siam_phyC_R         ---------- ---------- ---------- ---------- ---------- ---------- ---------- ---------- ---------- ----------

                                    210        220        230        240        250        260        270        280        290        300
                               ....|....| ....|....| ....|....| ....|....| ....|....| ....|....| ....|....| ....|....| ....|....| ....|....|
sp|O66037|PHYT_BACSD 3-phytase VRAFKMNSQT EGMAADDEYG SLYIAEEDEA IWKFSAEPDG GSNGTVIDRA DGRHLTPDIE GLTIYYAADG KGYLLASSQG NSSYAIYERQ GQNKYVADFQ
tr|A0A2K9KG41|A0A2K9KG41_9BACI VRAFKMNSQT EGMAADDEYG SLYIAEEDEA IWKFSAEPDG GSNGTVIDRA DGRHLTPDIE GLTIYYAADG KGYLLASSQG NSSYAIYDRQ GQNKYVADFR
tr|A0A8F7V592|A0A8F7V592_BACAM VRAFKMNSQT EGMAADDEYG SLYIAEEDEA IWKFSAEPDG GSNGTVIDRA DGRHLTPDIE GLTIYYAADG KGFLLASSQG NSSYAIYERQ GQNKYVADFQ
tr|Q8VQS1|Q8VQS1_BACAM Phytase VRAFKMNSQT EGMAADDEYS RLYIAEEDEA IWNFSAEPDG GSNGTVFDRA DGRHLTPDIE GLTIYYAADG KGYLMASSQG NNSYAIYDRQ GKNKYVADFR
Pr_B.amylo/siam_phyC_F         ---------- ---------- ---------- ---------- ---------- ---------- ---------- ---------- ---------- ----------
Pr_B.amylo/siam_phyC_R         ---------- ---------- ---------- ---------- ---------- ---------- ---------- ---------- ---------- ----------

                                    310        320        330        340        350        360        370        380
                               ....|....| ....|....| ....|....| ....|....| ....|....| ....|....| ....|....| ....|....| ...
sp|O66037|PHYT_BACSD 3-phytase ITDGPETDGT SDTDGIDVLG FGLGPEYPFG LFVAQDGENI DHGQKANQNF KMVPWERIAD KIGFHPQVNK QVDPRKMTDR SGK
tr|A0A2K9KG41|A0A2K9KG41_9BACI ITDGPETDGT SDTDGIDVLG FGLGPKYPFG LFVAQDGENI DHGQKANQNF KIVPWERIAD KIGFRPQVNK QVDPRKLTDR SGK
tr|A0A8F7V592|A0A8F7V592_BACAM ITDGPETDGT SDTDGIDVLG FGLGPEYPFG LFVAQDGENI DHGQKANQNF KMVPWERIAD KIGFHPQVNK QVDPRKLTDR SGK
tr|Q8VQS1|Q8VQS1_BACAM Phytase ITDGPETDGT SDTDGIDVLG FGLGREYPFG IFVAQDGENI DHRQKANQNF KIVPWERIAD QIGFRPLANE QVDPRKLTDR SGK
Pr_B.amylo/siam_phyC_F         ---------- ---------- ---------- ---------- ---------- ---------- ---------- ---------- ---
Pr_B.amylo/siam_phyC_R         ---------- ---------- ---------- ---------- ---------- ---------- ---------- -----KLTDR SGK
```

Figure S8 – Alignment of amino acid sequences of the PhyD second isoform. Pr_B.amylo/siam_phyC F and Pr_B.amylo/siam phyC_R are the locations of the universal primers at the terminal highly conserved regions of the amino acid sequence.

```
                                     10         20         30         40         50         60         70         80         90        100
                               ....|....| ....|....| ....|....| ....|....| ....|....| ....|....| ....|....| ....|....| ....|....| ....|....|
tr|A0A0G2UMU5|A0A0G2UMU5_BACLI MNFYKTLALS TLAASLWSPS WSSLPHNEAA AHKEFTVTAD AETEPVDTPD DAADDPAIWV HPKQPEKSRL ITTNKKSGLI VYDLKGKQLA AYPFGKLNNA
tr|Q8KTX7|Q8KTX7_BACLI Phytase MNFYKTLALS TLAASLWSPS WSSLPHNEAA AHKEFTVTAD AETEPVDTPD DAADDPAIWV HPKQPEDSRL ITTNKKSGLI VYDLKGKQLA AYPFGKLNNV
Pr_B.lichen_phyC_F             MNFYKTLALS ---------- ---------- ---------- ---------- ---------- ---------- ---------- ---------- ----------
Pr_B.lichen_phyC_R             ---------- ---------- ---------- ---------- ---------- ---------- ---------- ---------- ---------- ----------

                                    110        120        130        140        150        160        170        180        190        200
                               ....|....| ....|....| ....|....| ....|....| ....|....| ....|....| ....|....| ....|....| ....|....| ....|....|
tr|A0A0G2UMU5|A0A0G2UMU5_BACLI DLRYNFPLDG KKIDIAGASN RSDGKNTVEI YAFDGEKSKL KNIVNPQKPI QTDIQEVYGF SLYHSQKTGK FYALVTGKNG EFEQYELFDN GKGQVEGKKV
tr|Q8KTX7|Q8KTX7_BACLI Phytase DLRYNFPLDG KKIDIAGASN RSDGKNTVEI YAFDGEKSKL KNIVNPQKPI QTDIQEVYGF SLYHSQKTGK FYAMVTGKNG EFEQYELFDN GKGQVEGKKV
Pr_B.lichen_phyC_F             ---------- ---------- ---------- ---------- ---------- ---------- ---------- ---------- ---------- ----------
Pr_B.lichen_phyC_R             ---------- ---------- ---------- ---------- ---------- ---------- ---------- ---------- ---------- ----------

                                    210        220        230        240        250        260        270        280        290        300
                               ....|....| ....|....| ....|....| ....|....| ....|....| ....|....| ....|....| ....|....| ....|....| ....|....|
tr|A0A0G2UMU5|A0A0G2UMU5_BACLI RSFKMSSQTE GLAADDEYGK MYIAEEDVAI WSFSAEPDGG DKGKIVDRAD GPHLTADIEG LTIYYGEDGE GYLIASSQGD NRYAIYDRRG KNDYVTAFSI
tr|Q8KTX7|Q8KTX7_BACLI Phytase RSFKMSSQTE GLAADDEYGK MYIAEEDVAI WSFSAEPDGG DKGKIVDRAD GPHLTSDIEG LTIYYGEDGE GYLIASSQGD DRYAIYDRRG KNDYVTAFSI
Pr_B.lichen_phyC_F             ---------- ---------- ---------- ---------- ---------- ---------- ---------- ---------- ---------- ----------
Pr_B.lichen_phyC_R             ---------- ---------- ---------- ---------- ---------- ---------- ---------- ---------- ---------- ----------

                                    310        320        330        340        350        360        370        380
                               ....|....| ....|....| ....|....| ....|....| ....|....| ....|....| ....|....| ....|....| .
tr|A0A0G2UMU5|A0A0G2UMU5_BACLI EDGKEIDGTS DTDGIDVIGF GLGKTYPYGI FVAQDGENTE NGQPANQNFK IVSWEKIADA LDDKPDIDDQ VNPRKLKNRA K
tr|Q8KTX7|Q8KTX7_BACLI Phytase EDGKEIDGTS DTDGIDVIGF GLGKTYPYGI FVAQDGENTE NGQPANQNFK IVSWEKIADA LDDKPDIDDQ VDPRKLKNRA K
Pr_B.lichen_phyC_F             ---------- ---------- ---------- ---------- ---------- ---------- ---------- ---------- -
Pr_B.lichen_phyC_R             ---------- ---------- ---------- ---------- ---------- ---------- ---------- ----KLKNRA K
```

Figure S9. Alignment of amino acid sequences of the PhyD second isoform. Pr_B.lichen_phyC_F and Pr_B.lichen_phyC_R are the locations of the universal primers at the terminal highly conserved regions of the amino acid sequence.

```
                                       10         20         30         40         50         60         70         80         90         100
                               ....|....| ....|....| ....|....| ....|....| ....|....| ....|....| ....|....| ....|....| ....|....| ....|....|
PEY43863.1 3-phytase [Bacillus MLKEIATVAL MTSVLFSTVN STPDLVEKFP NSSVQEKQKH SKLVKVEAKE ETDAVANAGD AADDPAIWVH PHDSEKSKII GTNKEGGISI YNLKGKQLYS
WP_098335196.1 phytase [Bacill MLKEIATVAL MTSVLFSTVN STPDLVEKFP NSSVQEKQKH SKLVKVEAKE ETDAVANAGD AADDPAIWVH PHDSEKSKII GTNKEGGISI YNLKGKQLYS
Pr_B.cereus_3-phy_F            MLKEIATVAL ---------- ---------- ---------- ---------- ---------- ---------- ---------- ---------- ----------
Pr_B.cereus_3-phy_R            ---------- ---------- ---------- ---------- ---------- ---------- ---------- ---------- ---------- ----------

                                      110        120        130        140        150        160        170        180        190        200
                               ....|....| ....|....| ....|....| ....|....| ....|....| ....|....| ....|....| ....|....| ....|....| ....|....|
PEY43863.1 3-phytase [Bacillus YNFGKLNNVD VRYGFPIEGK KIDIAAASNR S--TNTIDIF AINPKTGELE NIMG--SPIQ SSMKEVYGFS LYHSQKTGIF YALVVGKDGT FEQYELFDNG
WP_098335196.1 phytase [Bacill YNFGKLNNVD VRYGFPIEGK KIDIAAASNR S--TNTIDIF AINPKTGELE NIMG--SPIQ SSMKEVYGFS LYHSQKTGIF YALVVGKDGT FEQYELFDNG
Pr_B.cereus_3-phy_F            ---------- ---------- ---------- ---------- ---------- ---------- ---------- ---------- ---------- ----------
Pr_B.cereus_3-phy_R            ---------- ---------- ---------- ---------- ---------- ---------- ---------- ---------- ---------- ----------

                                      210        220        230        240        250        260        270        280        290        300
                               ....|....| ....|....| ....|....| ....|....| ....|....| ....|....| ....|....| ....|....| ....|....| ....|....|
PEY43863.1 3-phytase [Bacillus KGKIEGKKVR ELKLSSQSEG IVADDEYGTI YIGEEDVAIW KFNAEPDGGN QPIAKVDGAD GNHLTADIEG LTIYYGKNGT GYLIASSQGN NSYAVYDRKG
WP_098335196.1 phytase [Bacill KGKIEGKKVR ELKLSSQSEG IVADDEYGTI YIGEEDVAIW KFNAEPDGGN QPIAKVDGAD GNHLTADIEG LTIYYGKNGT GYLIASSQGN NSYAVYDRKG
Pr_B.cereus_3-phy_F            ---------- ---------- ---------- ---------- ---------- ---------- ---------- ---------- ---------- ----------
Pr_B.cereus_3-phy_R            ---------- ---------- ---------- ---------- ---------- ---------- ---------- ---------- ---------- ----------

                                      310        320        330        340        350        360        370        380        390
                               ....|....| ....|....| ....|....| ....|....| ....|....| ....|....| ....|....| ....|....| ....|....| ....|.
PEY43863.1 3-phytase [Bacillus NNEYIGNFAI -VDGKN-TDG TSDTDGIDVM SFGLGEKYPN GIFLAQDGEN MDHGKIVNQN FKMVDWKKIA KGFDSRLQAE NNVNPRKLKY REILSK
WP_098335196.1 phytase [Bacill NNEYIGNFAI -VDGKN-TDG TSDTDGIDVM SFGLGEKYPN GIFLAQDGEN MDHGKIVNQN FKMVDWKKIA KGFDSRLQAE NNVNPRKLKY REILSK
Pr_B.cereus_3-phy_F            ---------- ---------- ---------- ---------- ---------- ---------- ---------- ---------- ---------- ------
Pr_B.cereus_3-phy_R            ---------- ---------- ---------- ---------- ---------- ---------- ---------- ---------- -------LKY REILSK
```

Figure S10. Alignment of amino acid sequences of the PhyD fourth isoform. Pr_B.cereus_3-phy_F ./ Pr_B.cereus_3-Phy_R are the locations of the universal primers at the terminal highly conserved regions of the amino acid sequence.

```
                                       10         20         30         40         50         60         70         80         90         100
                               ....|....| ....|....| ....|....| ....|....| ....|....| ....|....| ....|....| ....|....| ....|....| ....|....|
AHM26864.1 phytase [Bacillus c MQVLPKLAVS HYQAPVIAQV QAIAQTLPAD RIGDAADDPA IWVNHQHPEQ SRVLGTDKRG ALEVYDLNGQ RLQRLAVGRV NNVDVRQGFR LGGKTVDIAT
Pr_B.cereus_phy_F              MQVLPKLAV- ---------- ---------- ---------- ---------- ---------- ---------- ---------- ---------- ----------
Pr_B.cereus_phy_R              ---------- ---------- ---------- ---------- ---------- ---------- ---------- ---------- ---------- ----------

                                      110        120        130        140        150        160        170        180        190        200
                               ....|....| ....|....| ....|....| ....|....| ....|....| ....|....| ....|....| ....|....| ....|....| ....|....|
AHM26864.1 phytase [Bacillus c ASHRDHNAIS VFAIAPDSGE VSLLGEVPTP LKDIYGLCMY QPQGQIQVFV NDKNGRVLQY RLDDNHGAIK GTLVRDFRVN TQPEGCVADD KRGRFFLGEE
Pr_B.cereus_phy_F              ---------- ---------- ---------- ---------- ---------- ---------- ---------- ---------- ---------- ----------
Pr_B.cereus_phy_R              ---------- ---------- ---------- ---------- ---------- ---------- ---------- ---------- ---------- ----------

                                      210        220        230        240        250        260        270        280        290        300
                               ....|....| ....|....| ....|....| ....|....| ....|....| ....|....| ....|....| ....|....| ....|....| ....|....|
AHM26864.1 phytase [Bacillus c DVGIWAFNAD DTQAPAGTLI AKVGPMLHAD VEGLALWQGE RPILVASSQG NDSYVAYSAL PPYQLLGRFR IGLNSEAGID GTSXTDGIDI TSLALGKAYP
Pr_B.cereus_phy_F              ---------- ---------- ---------- ---------- ---------- ---------- ---------- ---------- ---------- ----------
Pr_B.cereus_phy_R              ---------- ---------- ---------- ---------- ---------- ---------- ---------- ---------- ---------- ----------

                                      310        320        330
                               ....|....| ....|....| ....|....| ....
AHM26864.1 phytase [Bacillus c QGLLAVQDGR KRLPEQGQNF KLVPFDAVLK LLQQ
Pr_B.cereus_phy_F              ---------- ---------- ---------- ----
Pr_B.cereus_phy_R              ---------- ---------- -------VLK LLQQ
```

Figure S11. Alignment of amino acid sequences of the PhyD fifth isoform and of the universal primers at the terminal highly conserved regions of the amino acid sequence.

Table S1. Oligonucleotide primers for amplification of gene encoding phytase from *Bacillus species* and cloning into integration vector

Pair	Name primer	Nucleotide sequence 5′.............3′	PCR product	GeneBank/ UniProtKB access number	Figure
1	Pr_B.subtil_phy_F	ATGAAGGTTTCAAAAACAATGCTGCTA	1149	GeneBank: NC_000964, UniProtKB: P42094; GeneBank:NP_389861.1, UniProtKB: A0A6M3ZCL5; GenBank:MK467451.1, UniProtKB: A0A5B8YW95	S7
	Pr_B.subtil_phy_R	CTAGCCGTCAGAACGGTCTTTCAGCTT			
2	Pr_B. spirireni_phyC_F	ATGAAGGTTTCAAAAACAATGCTGCT			
	Pr_B. spirireni _phyC_R	CTAGCCATCAGAACGGTCCTTCAACT			
3	Pr_B.amylo/siam_phyC_F	ATGAATCATTCAAAAACACTTTTGTTAAC	1149	GenBank: U85968.1, UniPortKB: O66037; GenBank: AF453255: UniPortKB: Q8VQS1	S8
	Pr_B.amylo/siam_phyC_R	TTATTTTCCGCTTCTGTCGGTCAGTTTTC			
4	Pr_B.cereus_phy_F	ATGCAGGTGCTGCCCAAACTGGCGGTC	1149	GenBank: KF953809, AHM26864.1, UniPortKB: W8TPH5	S10
	Pr_B.cereus_phy_R	CTTATTGTTGGAGTAATTTCAGCACCG			
5	Pr_B. cereus_3-phy_F	ATGTTAAAGGAAATAGCAACAGTTGCTTTA	1149	GenBank: PEY43863.1 GenBank: WP_098335196	S11
	Pr_B.cereus_3-phy_R	CTATTTGCTAAGTATTTCTCTATATTTTAG			
6	Pr_B.lichen_phyC_F	ATGAACTTTTACAAAACGCTCGCTTTATCA	1149	GenBank: KP893103, UniPortKB; A0A0G2UMU5 GenBank: AF469936, UniPortKB: Q8KTX7	S9
	Pr_B.lichen_phyC_F	TCCTTATTTGGCTCGGTTTTTCAGTTTTCG			
7	Pr-Phy-Bs/168/siamensis-f	CATTATGTGAATGAGGAACATCATTTCAA	1149	GeneBank: NC_000964, UniProtKB: P42094; GeneBank:NP_389861.1, UniProtKB: A0A6M3ZCL5; GenBank:MK467451.1, UniProtKB: A0A5B8YW95	S7
	Pr-Phy-Bs/168/siamensis–r	CTAGCCGTCAGAACGGTCTTTCAGCTTCC			
8	Pr-Phy-Bs/168/siamensis-f_BamHI	TTGGATCCATGGGACATTATGTGAATGAG	1071		
	Pr-Phy-Bs/168/siamensis_NotI	GGGCGGCCGCCTAGCCGTCAGAACGGTCT			
9	M13 fwd	GTAAAACGACGGCCAGT	1809	For construct PUV-3LT [29]	S12
	Pr-Vdac-Forw	GGTGTACACACATGCATATAGGATACATTT		For construct PUV-3LT [29]	

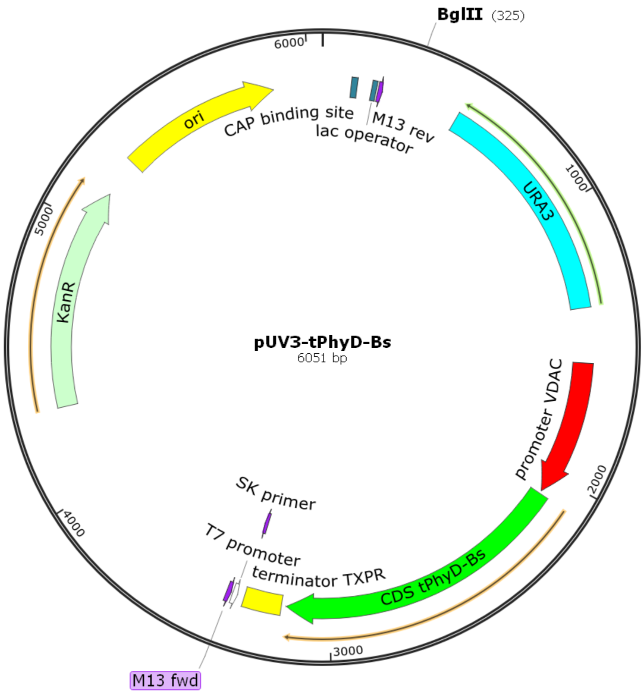

Figure S12. Map of the Integrative Vector pUV3-tPhyD-Bs for Expression of *Bacillus* Phytases (tPhyD-Bs-1, tPhyD-Bs-2) Under the VDAC Promoter of *Y. lipolytica* (Software SnapGene Viewer (5.0.7)).

1 2 3 4

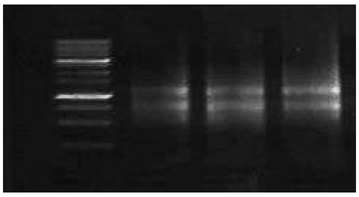

Figure S13 - Electrophoresis in 1.2% agarose gel. Lanes: 1) Mr - 1kb DNA marker, 2) total RNA from the biomass of *Y. lipolytica* PO1f (MatA, leu2-270, ura3- 302, xpr2-322, asp-2) , 3) total RNA from *Y. lipolytica* PO1f transformant biomass (pUV3-tPhyD-Bs-1)_5, 4) total RNA from *Y. lipolytica* PO1f transformant biomass (pUV3-tPhyD-Bs-1)_6.

1 2 3 4 5 6 7 8 9

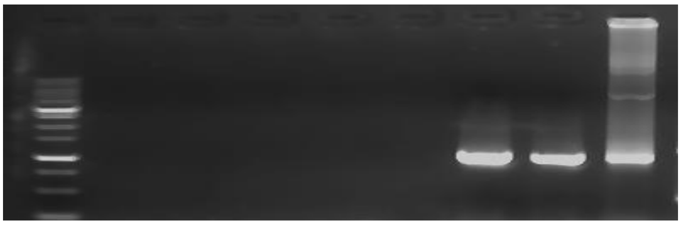

Figure S14 - Electrophoresis in 1.2% agarose gel. The result of cDNA amplification of the tPhyD-Bs-1 gene by the primer pair Pr-Phy-Bs/168/siamensis-f_BamHI and Pr-Phy-Bs/168/siamensis_NotI. Lanes: **1**) DNA marker Mr - 1kb**, 2**) only PCR mix, **3**) RNA from PO1f (MatA, leu2-270, ura3- 302, xpr2-322, asp-2), **4**) RNA from transformant Y. lipolytica PO1f (pUV3-tPhyD-Bs-1)_5, **5)** RNA from transformant Y. lipolytica PO1f (pUV3-tPhyD-Bs-1)_5., **6)** cDNA-PO1f (MatA, leu2-270, ura3- 302, xpr2-322, asp-2), **7)** cDNA transformant Y. lipolytica PO1f (pUV3-tPhyD-Bs-1)_5, **8)** vector pUV3-tPhyD-Bs-1.

1 2 3 4

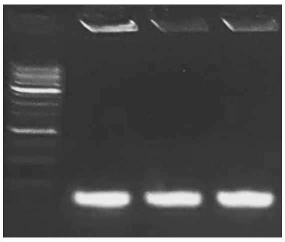

Figure S15 - Electrophoresis in 1.2% agarose gel. The result of amplification of ACT1 gene c cDNA. Lanes: 1) DNA marker Mr - 1kb, 2) transformant *Y. lipolytica PO1f* (pUV3-tPhyD-Bs-1)_5, 3) transformant Y. lipolytica PO1f (pUV3-tPhyD-Bs-1)_6, 4) PO1f (MatA, leu2-270, ura3- 302, xpr2-322, asp-2).

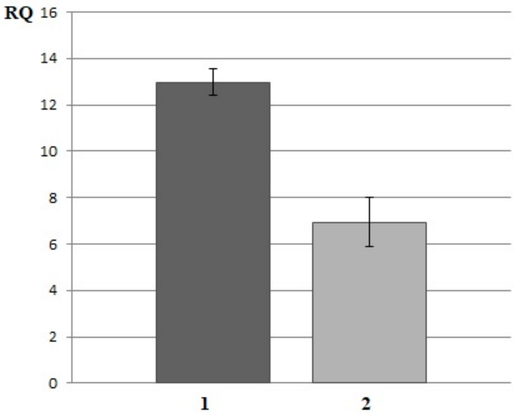

Figure S16 – Expression of the gene encoding phytase (tPhy-Bs-1 isoform) in transformants. RQ - relative quantity of transcripts of quantity. 1. transformant Y. lipolytica PO1f (pUV3-tPhyD-Bs-1)_5, 2. transformant *Y. lipolytica PO1f* (pUV3-tPhyD-Bs-1)_6. Experiments were performed in triplicate, data are presented as mean ± SD
